# Disruption of rich club organisation in cerebral small vessel disease

**DOI:** 10.1002/hbm.23479

**Published:** 2016-12-09

**Authors:** Anil M. Tuladhar, Andrew Lawrence, David. G. Norris, Thomas R. Barrick, Hugh S. Markus, Frank‐Erik de Leeuw

**Affiliations:** ^1^ Department of Neurology Radboud University Medical Center, Donders Institute for Brain, Cognition and Behaviour Nijmegen The Netherlands; ^2^ Centre for Cognitive Neuroimaging Radboud University, Donders Institute for Brain, Cognition and Behaviour Nijmegen The Netherlands; ^3^ Department of Clinical Neurosciences, Neurology Unit University of Cambridge Cambridge United Kingdom; ^4^ Erwin L. Hahn Institute for Magnetic Resonance Imaging, University of Duisburg‐Essen Arendahls Wiese 199, Tor 3 Essen D‐45141 Germany; ^5^ MIRA Institute for Biomedical Technology and Technical Medicine, University of Twente Enschede The Netherlands; ^6^ St. George's University of London, Neuroscience Research Centre, Cardiovascular and Cell Sciences Research Institute London United Kingdom

**Keywords:** cerebral small vessel disease, structural networks, diffusion tensor imaging, graph‐theory, rich club organisation

## Abstract

Cerebral small vessel disease (SVD) is an important cause of vascular cognitive impairment. Recent studies have demonstrated that structural connectivity of brain networks in SVD is disrupted. However, little is known about the extent and location of the reduced connectivity in SVD. Here they investigate the rich club organisation—a set of highly connected and interconnected regions—and investigate whether there is preferential rich club disruption in SVD. Diffusion tensor imaging (DTI) and cognitive assessment were performed in a discovery sample of SVD patients (*n =* 115) and healthy control subjects (*n =* 50). Results were replicated in an independent dataset (49 SVD with confluent WMH cases and 108 SVD controls) with SVD patients having a similar SVD phenotype to that of the discovery cases. Rich club organisation was examined in structural networks derived from DTI followed by deterministic tractography. Structural networks in SVD patients were less dense with lower network strength and efficiency. Reduced connectivity was found in SVD, which was preferentially located in the connectivity between the rich club nodes rather than in the feeder and peripheral connections, a finding confirmed in both datasets. In discovery dataset, lower rich club connectivity was associated with lower scores on psychomotor speed (*β* = 0.29, *P* < 0.001) and executive functions (*β* = 0.20, *P* = 0.009). These results suggest that SVD is characterized by abnormal connectivity between rich club hubs in SVD and provide evidence that abnormal rich club organisation might contribute to the development of cognitive impairment in SVD. *Hum Brain Mapp 38:1751–1766, 2017*. © **2017 Wiley Periodicals, Inc.**

## INTRODUCTION

Cerebral small vessel disease (SVD) is an important cause of vascular cognitive impairment and vascular dementia [Roman et al., 2002]. The cognitive consequences of SVD are distinct from Alzheimer's type dementia with relative preservation of memory in the presence of deficits of executive function and processing speed [Roman et al., 2002; Zhou and Jia, 2009]. The pathophysiology of these deficits is still incompletely understood. SVD is characterized by damage to the white and deep grey matter structures of the brain, primarily appearing as white matter hyperintensities (WMH) and lacunes of presumed vascular origin [Wardlaw et al., 2013]. For a number of years there has been considerable interest in diffusion‐weighted magnetic resonance imaging (DW‐MRI), which appears sensitive to the impact of WMH and lacunes, and identifies abnormalities in white matter appearing normal on T2 weighted sequences [Holtmannspotter et al., 2005; Schmidt et al., 2010; Van Norden et al., 2012]. Structural networks, constructed from DW‐MRI, provide a measure of whole brain connectivity and its disruption has found to be associated with the traditional MRI markers of SVD (WMH, lacunes and microbleeds) [Lawrence et al., 2014; Tuladhar et al., 2015b]. In addition, network disruption mediates, at least in part, the association between these markers and cognitive dysfunction in SVD. As a result structural networks have been proposed as a disease marker for SVD [Lawrence et al., 2014; Reijmer et al., 2015; Tuladhar et al., 2015b, 2016].

Brain networks are comprised of a few selective central regions with a high number of connections (i.e., hub nodes) that also show evidence of ‘rich club’ properties. This refers to the presence of a clique of highly connected nodes (i.e., nodes ‘rich’ in connections) that furthermore strongly connect to each other, often over physically long distances [Van den Heuvel et al., 2012]. They form a resilient backbone to the network which supports efficient communication in the brain [Van den Heuvel et al., 2012]. Such rich club organisation is a feature of brain networks measured in a wide variety of circumstances, including: different modalities (structural connectivity [Van den Heuvel and Sporns, 2011], functional connectivity [Sasai et al., 2014]), different developmental stages (newborn human brain [Ball et al., 2014], mature human brain [Van den Heuvel and Sporns, 2011]), different species (macaque [Harriger et al., 2012], cat [De Reus and van den Heuvel, 2013b]) and different spatial and temporal scales (from single neuron‐to‐neuron [Teller et al., 2014] to cellular scale [Towlson et al., 2013]). The near ubiquity and centrality of rich clubs in the brain network has led to interest in how the rich club is affected by disease [Crossley et al., 2014]. It is, however, unknown whether damage to the white matter affected by SVD would result in widespread or more localized network disruption involving primarily connections between the highly connected nodes.

In this article we analyse rich clubs in patients with SVD to increase our understanding of the SVD‐related pathophysiology. Building on the previous work showing reduced network efficiency in SVD which correlates with cognitive impairment [Lawrence et al., 2014; Tuladhar et al., 2015b], we hypothesize that the pattern of reduced white matter connectivity in SVD will show a preferential reduction of the connections between rich club nodes due to the location of the SVD‐related lesions. To this end, we investigated the structural networks in a cohort of SVD patients and control subjects using diffusion tensor imaging and whole‐brain tractography. To replicate the findings, a second independent dataset of SVD patients and control subjects was included.

## METHODS

### Study Population

Two datasets were included in this study. The discovery dataset comprised all baseline data from SVD patients (*n =* 115) enrolled in the St George's Cognition and Neuroimaging in Stroke (SCANS) study [Lawrence et al., 2013], along with a similarly aged population‐based control group (*n =* 50) recruited to the St. George's Neuropsychology and Imaging in Elderly (GENIE) study [Charlton et al., 2010], both imaged on the same MR system with the same MR protocols. SVD was defined as a clinical lacunar stroke syndrome with an anatomically corresponding lacunar infarct on MRI in addition to confluent white matter hyperintensities (total Fazekas score 2 or higher) [Fazekas et al., 1987] on T2‐weighted MRI. The replication dataset is a part of the ‘Radboud University Nijmegen Diffusion tensor and MRI Cohort’ (RUN DMC) study, a prospective study that was designed to investigate risk factors and cognitive, motor, and mood consequences of functional and structural brain changes as assessed by MRI among elderly with SVD, consisting of 503 participants with SVD on neuroimaging [Van Norden et al., 2011]. For this study, SVD patients (*n =* 49) with a phenotype identical to those patients in SCANS were recruited, that is, inclusion criteria were a clinical lacunar stroke syndrome as well as confluent white matter hyperintensities (total Fazekas score 2 or higher) [Fazekas et al., 1987] on T2‐weighted MRI. In addition a ‘control’ group of age‐ and sex‐matched participants with no history of stroke and a WMH Fazekas score lower than 2, were also recruited from the RUN DMC study (*n =* 108).

**Figure 1:**
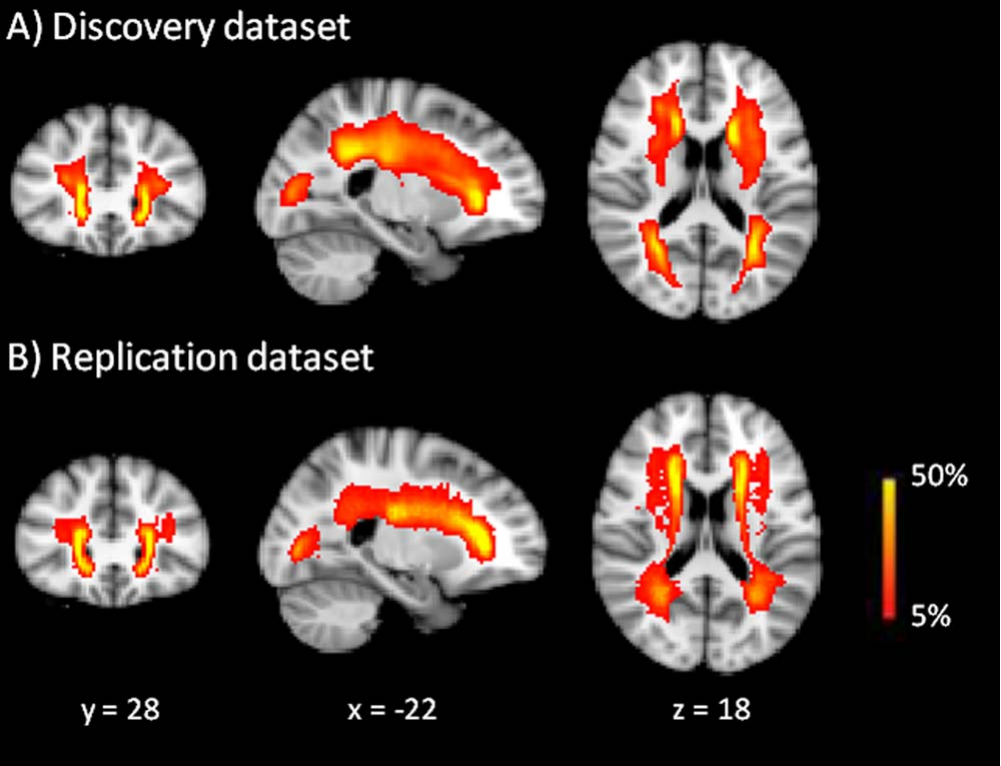
Probability maps of white matter hyperintensities. The probability distribution of white matter hyperintensities (in red) for the discovery (*n =* 115) and replication dataset (*n =* 49), thresholded from 5% to 50%. The images are projected onto spatially normalized (Montreal Neurological Institute stereotactic space). [Color figure can be viewed at http://wileyonlinelibrary.com]

The characteristics of the discovery dataset (*n =* 165) and the replication dataset (*n =* 157) are shown in Table 1. SVD load (WMH load and presence of lacunes) and the distribution of WMH were similar between the discovery and replication datasets (Fig. 1).

**Table 1:** Baseline characteristics for the discovery and replication dataset

	SCAN data discovery dataset		RUNDMC data replication dataset	
	Control group N = 50	SVD group N = 115	Control group N = 108	SVD group N = 49
Demographic				
Age, years (SD)	70.2 (9.3)	70.2 (9.7)	69.1 (6.0)	69.1 (8.6)
Gender, female (%)	21 (42)	39 (34)	50 (46)	21 (43)
MMSE	29 (25; 30)	28 (16; 30)	29 (27; 30)	27 (26; 29)
Vascular risk factors				
Hypertension, (%)	28 (49)	112 (93)	81 (75)	45 (92)
Diabetes, (%)	0 (0)	24 (20)	10 (9)	10 (20)
Smoking^a^, (%)	32 (56)	55 (46)	66 (61)	38 (78)
BMI, kg/m^2^	25.2 (3.9)	27.1 (4.9)	27.4 (4.1)	27.3 (3.9)
Neuroimaging				
WMH, mL (SD)	0.84 (1.2)	3.1 (2.6)	0.58 (0.4)	3.4 (2.4)
Lacune(s)^b^ (%)	18 (36.0)	86 (74.8)	12 (30.6)	33 (67.4)
GMV, mL (SD)	632.0 (54.4)	568.0 (71.6)	622.5 (62.8)	615.7 (77.7)
WMV, mL (SD)	434.0 (54.7)	434.0 (72.5)	457.8 (66.6)	452.0 (67.4)
TBV, mL (SD)	1066.1 (94.2)	1002.1 (115.7)	1080.3 (118.7)	1067.8 (130.0)

Data represent mean (standard deviation) or number (percentage) or median (range). Hypertension is defined as treatment with antihypertensive drugs or systolic BP ≥ 140 mm Hg or diastolic blood pressure ≥90 mm Hg.

a Smoking represents current and ex‐smokers.

b Lacune(s) represents number (percentage) of group with one or more cavitated lacunes on MRI.

MMSE, mini mental state examination; BMI, body mass index; WMH, white matter hyperintensities; GMV, grey matter volume; WMV, white matter volume; TBV, total parenchymal brain volume.

### Cognitive Performance

#### Discovery dataset

The results of the cognitive data is only reported for SVD patients because cognitive assessments used in the GENIE study from which the control group was obtained were not comparable. Testing was performed at least 3 months post‐stroke to minimise acute effects of stroke on performance. Tests comprised previously published, widely‐used tasks chosen to characterise reported cognitive impairment in SVD [Charlton et al., 2006]. Task performance was age‐scaled to normative data from the general population and indices summarising performance *z*‐scores across groups of related tasks were produced. Premorbid IQ was estimated using the National Adult Reading Test—Revised (NART‐R). NART‐R error scores were converted to estimated full‐scale IQ scores (NART‐IQ). Executive function and processing speed was calculated as previously described [Lawrence et al., 2013]: Executive Function (EF): Trail Making Test, modified Wisconsin Card Sorting Test, Phonemic Fluency. Processing Speed (PS): Digit Symbol Substitution, Speed of Information Processing Task, Grooved Pegboard Task.

#### Replication dataset

Psychomotor speed was calculated as the mean of the z‐scores of the 1‐letter subtask of the Paper‐Pencil Memory Scanning Task, the reading subtask of the Stroop test and the Symbol‐Digit Substitution Task [Tuladhar et al., 2015a]. Executive function was not available for this dataset.

### MRI Acquisition

#### Discovery dataset

MR images were acquired at St George's University of London using a 1.5T Signa HDxt MRI system (General Electric, Milwaukee, WI), which included an axial fluid‐attenuated inversion recovery (FLAIR), coronal spoiled gradient recalled echo 3‐dimensional T1‐weighted, axial single shot diffusion‐weighted spin echo planar imaging with isotropic voxels (2.5 mm^3^), 4 unweighted scans and 25 non‐collinear diffusion gradient directions at b = 1,000 s/mm^2^ in positive and negative diffusion gradient directions.

#### Replication dataset

MR images were acquired on a 1.5 Tesla Siemens Magneton Sonata scanner (Siemens Medical Solutions, Erlangen, Germany) and included T1‐weighted 3D magnetization‐prepared rapid gradient‐echo (MPRAGE) imaging, a FLAIR sequence and a DTI sequence (isotropic voxel size 2.5 mm^3^, 4 unweighted scans, 30 diffusion weighted scans at b = 900 s/mm^2^).

Full descriptions of acquisition protocols have been previously published: discovery [Lawrence et al., 2013] and replication dataset [Van Norden et al., 2011].

### Conventional Markers for SVD

#### Discovery dataset

Imaging markers for SVD used in this study for analyses were normalized brain volume, WMH and lacunes. Normalized brain volume is a measure of brain volume adjusted for the head size and was calculated on a T1‐weigthed image using SIENAX (FMRIB Software Library, FSL v4.1). WMH were manually segmented and lacunes were counted by a trained rater [Lawrence et al., 2013].

#### Replication dataset

Similarly, WMH were manually delineated and lacunes were counted by a trained rater. Brain volume was calculated as a sum of grey and white matter volume using automated segmentation procedure in SPM5 on a T1‐weighted image and normalized to the total intracranial volume to adjust for the head size [Tuladhar et al., 2015b].

### DTI Preprocessing

#### Discovery dataset

DW‐MRIs were corrected for eddy current distortions using FSL toolbox and DTI were calculated. Whole‐brain deterministic tractography was performed at super‐resolution (0.5 mm^3^) using in‐house software [Lawrence et al., 2014]. Streamlines were terminated when the angle between consecutive principal eigenvectors exceeded 45° or fractional anisotropy was less than 0.20.

#### Replication dataset

DW‐MRI was corrected for cardiac and head motion artefacts as well as eddy currents using ‘PATCH’ [Zwiers, 2010]. Whole‐brain deterministic tractography was based on fibre assignment by continuous tracking (FACT) method and applied using Diffusion Toolkit (http://www.trackvis.org). The tracking algorithm started at the centre of the voxels with fractional anisotropy greater than 0.15 and ended when the fibre tracks left the brain mask, encountered voxels with fractional anisotropy less than 0.15 or when the turning angle exceeded 60°. These turning angles and FA thresholds were used in the replication dataset in order to achieve similar number of constructed streamlines between the dataset due to the tractography at super‐resolution in the discovery dataset.

### Network Nodes

For both datasets, brain regions were parcellated in each subject using the Automatic Anatomical Label (AAL) template [Tzourio‐Mazoyer et al., 2002] into 90 regions, excluding the cerebellar regions. For each subject (both discovery and replication dataset), T1‐weighted images were first registered to non‐diffusion weighted image using FLIRT. In the discovery dataset, the T1‐weighted were then non‐linearly registered to Montreal Neurological Institute (MNI) 152 template using Advanced Normalization Tool (ANTS) [Avants et al., 2011]. In the replication dataset, non‐linear registration was conducted using FNIRT. Linear and non‐linear transformations were finally combined to register the AAL template to each subject's diffusion space.

### Network Edges

Two regions were considered connected if the endpoints of a tractography streamline were located within the pair of brain regions. Connection strengths were estimated based on a modified method of Hagmann and colleagues [Hagmann et al., 2007; Lawrence et al., 2014] and calculated as the sum of the inverse of the streamlines length, including a scaling factor to correct for the number of seeds per millimetre. Weighted edges were thresholded at 1, to reduce noise‐related false‐positive connections.

### Network Measures

Graph theoretical measures were calculated from the structural network using the Brain Connectivity Toolbox [Rubinov and Sporns, 2010]. These measures included: (1) node degree, representing the number of connections of a node; (2) network density, defined as the ratio between the number of connections present and the total number of possible connections in a network; (3) total network strength, computed as the sum of all connection strengths in a network; (4) efficiency, expressed as the inverse of the shortest path length between two nodes. Normalized efficiency was calculated by dividing the network efficiency of the networks with a set of random networks (*n =* 100) with the same size and degree distribution using Brain Connectivity Toolbox.

### Rich Club Measures

The rich club coefficient is measured as the ratio between the sum of the weights of the edges connecting a subset of nodes (exceeding a certain degree‐threshold) and the sum of the weights of the strongest connections of the total network. The rich club coefficients were then normalised by dividing by the averaged rich club coefficients of a set of random networks (with the same size and degree distribution). A network with a normalized rich club coefficient exceeding 1 is considered as a network with a rich club organisation. Rich club coefficients were estimated using weighted network connections [Van den Heuvel and Sporns, 2011].

**Figure 2:**
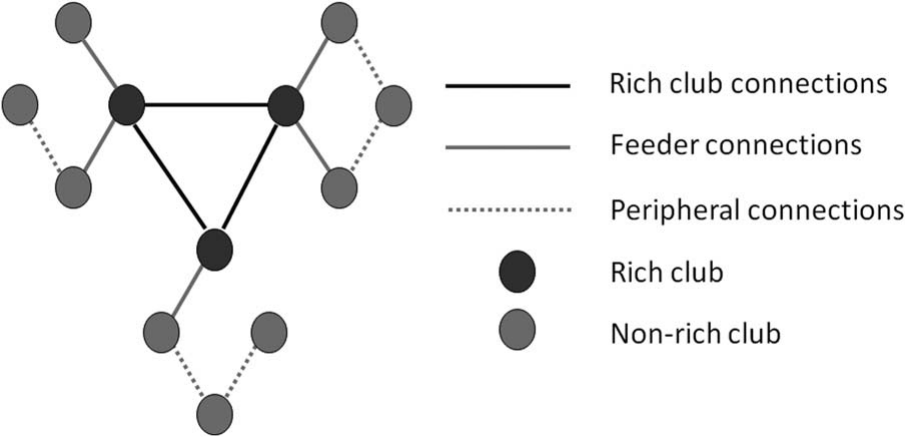
Rich club organisation. Nodes were classified as rich club, or non‐rich club. From this, connections linking two rich club nodes are rich club connections; connections linking a rich club node to a non‐rich club node are feeder connections; and connections between non‐rich club nodes are peripheral connections.

For analysis of the rich club in SVD we selected the top 8 highest degree nodes (top 9% of the nodes) averaged across both groups. The node degree was calculated by taking the mean observed average degree for each node across subjects from both groups. Other selection procedures of rich club nodes were also evaluated to test whether results were independent of the methods used: (1) defining the rich club nodes based on a group‐averaged network, comprising edges present in at least 30% of the group and (2) the selection of top 8 highest ranking degree nodes calculated per‐subject (such that rich club membership varied between individuals). The selection of top 8 highest ranking nodes was based on previous reports [Collin et al., 2014a; Van den Heuvel et al., 2013]. In each case the connections of the network were then classified for further analysis [Van den Heuvel et al., 2012; Van den Heuvel and Sporns, 2011]: connections between the rich club nodes were designated as rich club connections; connections to the rich club nodes as feeder connections and connections between the non‐rich club nodes as peripheral connections (Fig. 2). For each type of connection group: rich club, feeder and peripheral, a summary measure of connectivity the ‘connection strength’ was calculated as the sum of the edge weights for that group.

### Statistical Analyses

WMH volumes and the number of lacunes were log‐transformed to obtain a normal distribution. Differences in network measures between the control and SVD group were examined for statistical significance using Welch's independent *t*‐tests because of the unequal sample sizes. Since the control group was age‐ and sex‐matched, we did not additionally correct for age and sex. Permutation testing (10,000 iterations) was used to create a null distribution of the largest cluster size, which then used to calculate the family‐wise error p‐corrected value. Finally, multiple regression analyses were performed to examine the relation between rich club connectivity and cognitive functions, while adjusted for the effects of confounding factors (for the discovery dataset: age, sex and NART‐IQ; for the replication dataset: age, sex and education). To establish mediation, we performed Sobel tests, in which the *z*‐score represents a test of statistical significance for the indirect (mediated) relationship between the outcome and the potentially mediated variable given a potential mediator. Variance inflation factors were calculated to examine whether multicollinearity was present in the models. Multicollinearity was considered if the variance inflation factor was above five.

## RESULTS

### Network Measures

#### Discovery dataset

**Figure 3:**
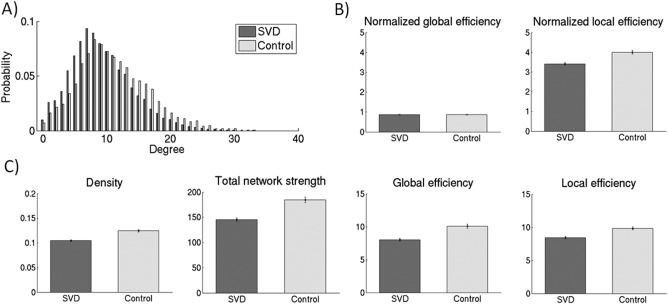
Network characteristics of the discovery dataset. (A) Degree distribution of the structural networks for the SVD (*n =* 115) and control group (*n =* 50), averaged across the groups. SVD networks showed more nodes with lower degree, whereas networks of the control group had more nodes with higher degree. (B) The networks of both the control and SVD group showed a small‐world topology, showing a high level of global integration (normalized global efficiency ≈ 1) and a high level of local clustering (normalized local efficiency > 1). (C) Networks of SVD patients showed significantly lower density, total network strength, global efficiency and local efficiency compared with network of the control group (*P* < 0.05). The figure depicts mean (standard error).

Both SVD and control networks showed a right skewed degree distribution (Fig. 3A), which is indicative of the presence of a small number of nodes with high connections. Patients with SVD had fewer high degree brain regions than controls (Fig. 3A). The following global network measures of the discovery dataset were previously reported [Lawrence et al., 2014]. The global measures are reported to verify the global changes and to compare those with the replication dataset for validation. The networks of control and SVD group had a small‐world architecture, showing a high level of local clustering and a high level of global integration (Fig. 3B): the local efficiency was higher than that for the random networks with similar degree distribution (normalized local efficiency > 1) and the global efficiency was comparable to that for the random networks (normalized global efficiency ≈ 1). The networks of SVD patients in the discovery dataset showed a lower density (*P* < 0.001, df = 106.5), total network strength (*P* < 0.001, df = 96.5), global efficiency (*P* < 0.001, df = 97.0) and local efficiency (*P* < 0.001, df = 105.2) compared with the networks of control group (Fig. 3C). Global efficiency was positively associated with processing speed (*β* = 0.36, *P* < 0.001) and executive function (*β* = 0.32, *P* < 0.001), adjusted for age, sex and NART‐IQ.

#### Replication dataset

**Figure 4:**
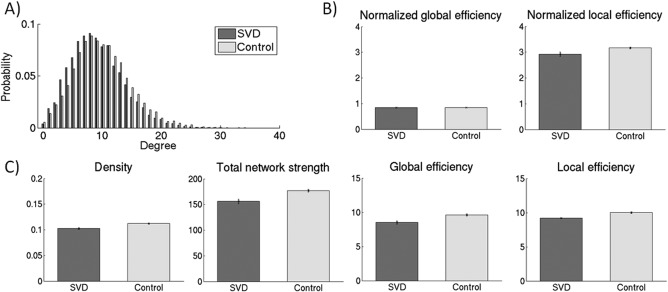
Network characteristics of the replication dataset. (A) Degree distribution of the structural networks for the high SVD (*n =* 49) and low SVD group (*n =* 108), averaged across the groups. B) The networks of both the control and SVD group showed a small‐world topology, showing a high level of global integration (normalized global efficiency ≈ 1) and a high level of local clustering (normalized local efficiency > 1). (C) Confirming the discovery dataset, networks of SVD group showed significantly lower density, total network strength, global efficiency and local efficiency compared with network of the control group (*P* < 0.05). The figure depicts mean (standard error).

Similar results were found for the replication dataset (Fig. 4). Both SVD and control group showed a right skewed degree distribution. Small‐world architecture was present in both groups (normalized local efficiency > 1 and normalized global efficiency ≈ 1). SVD group showed a lower density (*P* < 0.001, df = 78.2), total network strength (*P* < 0.001, df = 86.1), global efficiency (*P* < 0.001, df = 92.3) and local efficiency (*P* = 0.003, df = 75.6), compared with the control group. Global efficiency was associated with psychomotor speed (*β* = 0.32, *P* = 0.028), adjusted for age, sex and education.

### Reduced Rich Club Organisation in SVD Group

#### Discovery dataset

**Figure 5:**
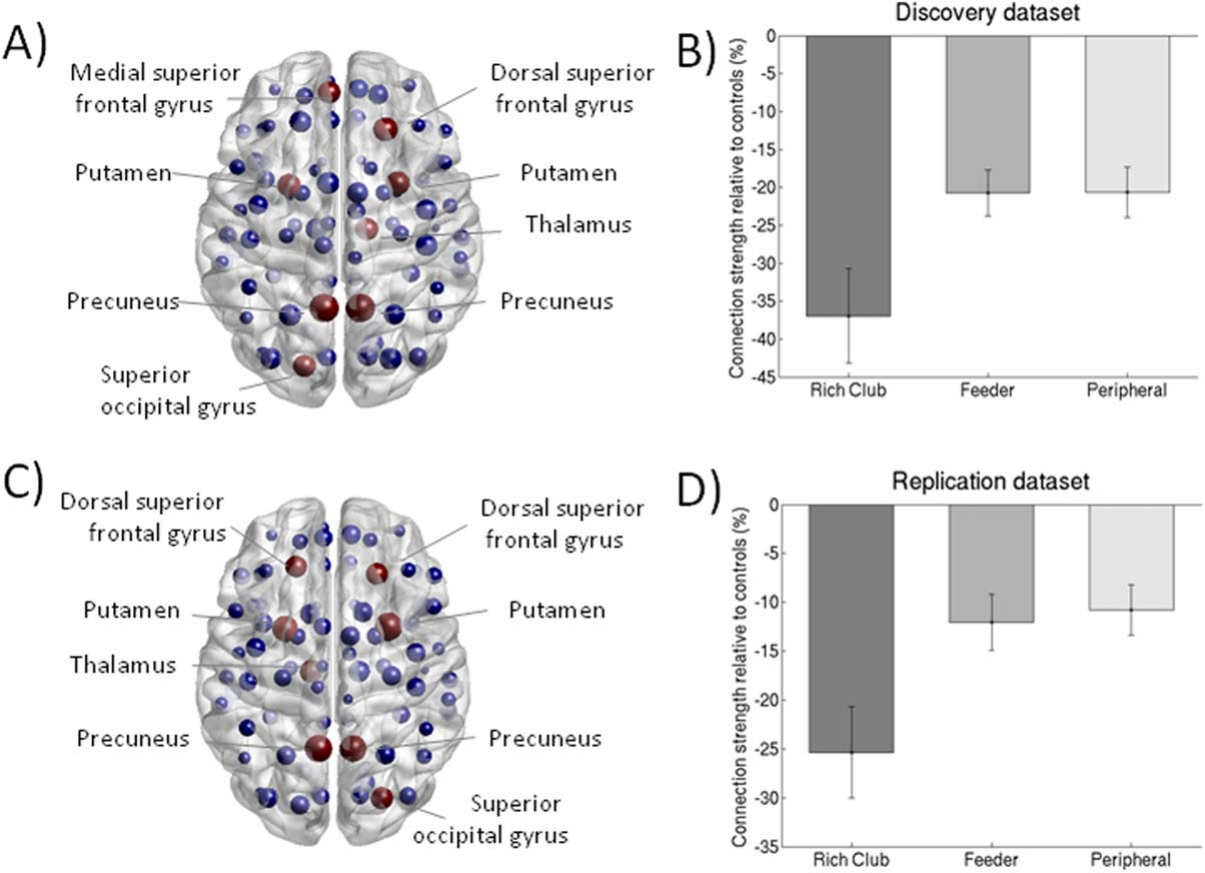
Rich club impairment in the discovery and replication dataset. Nodes in anatomical space with the rich club (highest 8 ranked nodes based on degree) depicted in red for the discovery dataset (A) and replication dataset (C); the size of each node is proportional to its degree. SVD group showed significantly lower connection strength of the rich club, feeder and peripheral connections compared with the control group, with a greater reduction in connection strength of the rich club connections for the discovery dataset (B) and replication dataset (D). The figure depicts mean (standard error). [Color figure can be viewed at http://wileyonlinelibrary.com]

The 8 nodes (≈9%) with the highest degree averaged across both groups were selected as the rich club nodes. The top 8 most connected brain regions of the discovery dataset were bilateral precuneus, bilateral putamen, left medial superior occipital gyrus, left medial superior frontal gyrus, right thalamus and right dorsal superior frontal gyrus (Fig. 5A). The percentage of rich club, feeder and peripheral connections to the total network connections is respectively 2.4%, 25.9% and 71.7%. Normalised rich club coefficients given this definition were significantly greater than 1 for both groups at this threshold, with significant group differences such that the coefficients were lower in the SVD group (1.74 ± 0.72) than in the control group (1.99 ± 0.75) (*P* = 0.022; 10,000 permutations). SVD patients showed 37.0% reduction in the connectivity strength of the rich club connections (*P* < 0.001, df = 81.5), 20.7% reduction in feeder and peripheral connections (*P* < 0.001, df = 102.0; *P* < 0.001, df = 94.3, respectively) relative to the control groups (Fig. 5B). To account for the overall connectivity strength across the groups, additional analyses were performed while adjusting for the total network strength [Van den Heuvel et al., 2013]. The reduction in the connection strength of the rich club connections remained significant (*P* = 0.002, df = 86.5), whereas the reductions for the feeder and peripheral connection strengths were not significant. Also, these differences remained significant after controlling for brain volume (to account for brain atrophy).

Given the differential reduction in rich club connection strength, we tested for group differences in the ratio of rich‐club to feeder and of rich club to peripheral connection strengths [Van den Heuvel et al., 2013]. Compared with the control group, SVD patients showed significantly lower ratios for rich club/feeder (*P* < 0.001, df = 84.5) and rich club/peripheral connection (*P* < 0.001, df = 87.1).

The results were comparable when using the streamline count normalized for the ROI volume as the weighting procedure for edges. SVD patients showed 41.4% reduction in the connectivity strength of the rich club connections (*P* < 0.001, df = 73), 30.6% reduction in feeder (*P* < 0.001, df = 79) and 25% reduction in peripheral connections (*P* < 0.001, df = 87). Compared with the control group, SVD patients showed significantly lower ratios for rich club/feeder (*P* < 0.001) and rich club/peripheral connection (*P* < 0.001).

Additional analyses were performed using different selection methods of rich club nodes to examine the robustness of the results independent of the methods used. Similar results were found when the rich club nodes were selected on basis of the group‐average network: 27.5% reduction in rich club connections (*P* < 0.001), 22.9% reduction in feeder connections (*P* < 0.001) and 20.6% reduction in peripheral connections relative to the group (*P* < 0.001). In addition, selecting the rich club nodes on basis of individual networks, set at the threshold of top ≈9% of the highest degree nodes (equivalent to 8 rich club nodes) showed similar results: 26.9% reductions in rich club connections (*P* < 0.001), 20.5% in feeder connections (*P* < 0.001) and 21.3% in peripheral connections (*P* < 0.001).

**Figure 6:**
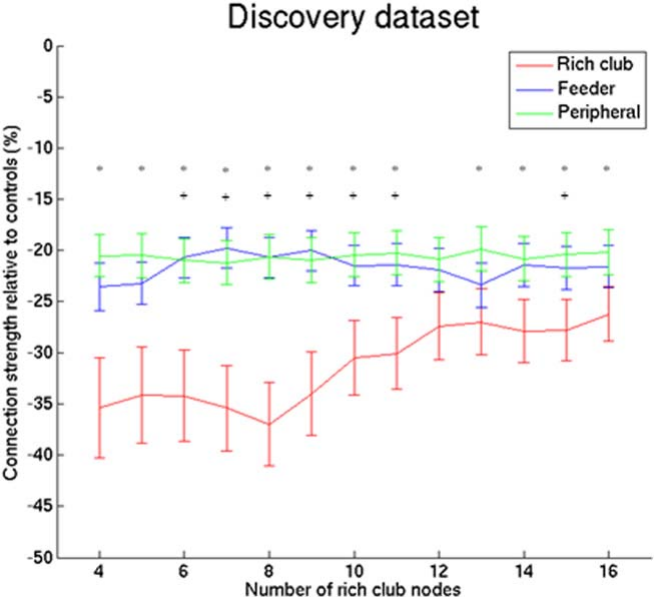
Rich club impairment in the discovery dataset at different number of rich club nodes. SVD group showed lower connection strength of the rich club, feeder and peripheral connections compared with the control group (*P* < 0.001 for all number of rich club nodes) with a greater reduction in connection strength of the rich club connections. SVD group showed significantly lower ratios for rich club/feeder and rich club/peripheral connection at 6–11 and 15 rich club nodes. Error bars denote standard error. ^+^ *P* < 0.05 (Bonferroni corrected) for the ratio between rich club and feeder connections. *P < 0.05 (Bonferroni corrected) for the ratio between rich club and peripheral connections. [Color figure can be viewed at http://wileyonlinelibrary.com]

In addition, we re‐analysed the rich club organisation at different number of rich club nodes. These results showed that reduction in rich club/feeder/peripheral connections relative to controls are comparable to the selection of 8 rich club nodes (Fig. 6).

#### Replication dataset

The top 8 most connected brain regions of the replication dataset were bilateral precuneus, bilateral putamen, bilateral dorsal superior frontal gyrus, right medial superior occipital gyrus and left thalamus left (Fig. 5C), comparable to the discovery dataset. In the replication dataset, the rich club organisation was present in both groups. The SVD group (1.58 ± 0.55) tended to have a lower normalized rich club coefficient than the control group (1.67 ± 0.64; *P* = 0.10, 10,000 permutations). The SVD group showed 25.4% reduction in rich club connections relative to the control group (*P* < 0.001, df = 110.3), 12.0% reduction in feeder connections (*P* = 0.001, df = 88.4) and 10.8% reduction in peripheral connections (*P* = 0.001, df = 87.8) (Fig. 5D). Ratios for rich club/feeder and rich club/peripheral connections were significantly reduced in SVD group (*P* = 0.013, df = 107.0; *P* = 0.007, df = 97.6, respectively).

### Effects of Fibre Length

**Figure 7:**
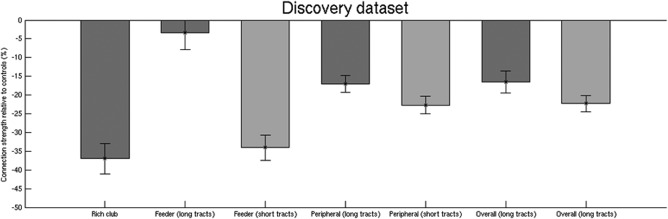
Connection strength of the short and long tracts of feeder and peripheral connections in the discovery dataset. The short rather than long tracts of both feeder and peripheral showed a greater reduction of connection strength relative to controls. This observation is evidence that the vulnerability of the rich club to small vessel disease effects is independent of the longer fibre length found in the rich club.

Rich club connections are on average physically longer than feeder or peripheral connections [Van den Heuvel et al., 2012]. The vulnerability of the rich‐club may simply arise from its longer length connections. To address this, we dichotomized both the feeder and peripheral connections into groups with long and short length connections using a median split based on fibre length. The average length of the long tracts of the feeder and peripheral connections are longer than the average fibre length of the rich club connections (Table 2). The effects of SVD on tracts of different length were estimated by calculating the connection strength relative to controls for each length group (Fig. 7). For both feeder and peripheral connections, the short rather than long tracts showed a greater influence of SVD (i.e., a greater reduction in connection strength relative to controls). This effect was significant for feeder connections (*P* < 0.001), and marginally significant for peripheral connections (*P* = 0.077). These results suggest that the vulnerability of the rich club to SVD effects is independent of the longer fibre length found in the rich club.

**Table 2:** Average fibre length of each connection group

	Rich club connections (not dichotomized)	Feeder connections	Peripheral connections
Average fibre length	85.0 mm	73.1 mm	62.0 mm
Short tracks	–	40.3 mm	38.5 mm
Long tracts	–	103.1 mm	95.9 mm

The feeder and peripheral connections were dichotomized into groups with long and short length connections using a median split based on fibre length. The average length of the long feeder and peripheral tracts are higher than the average fibre length of the rich club connections.

### Relation between Rich Club Connection Strength and Cognitive Function

#### Discovery dataset

**Figure 8:**
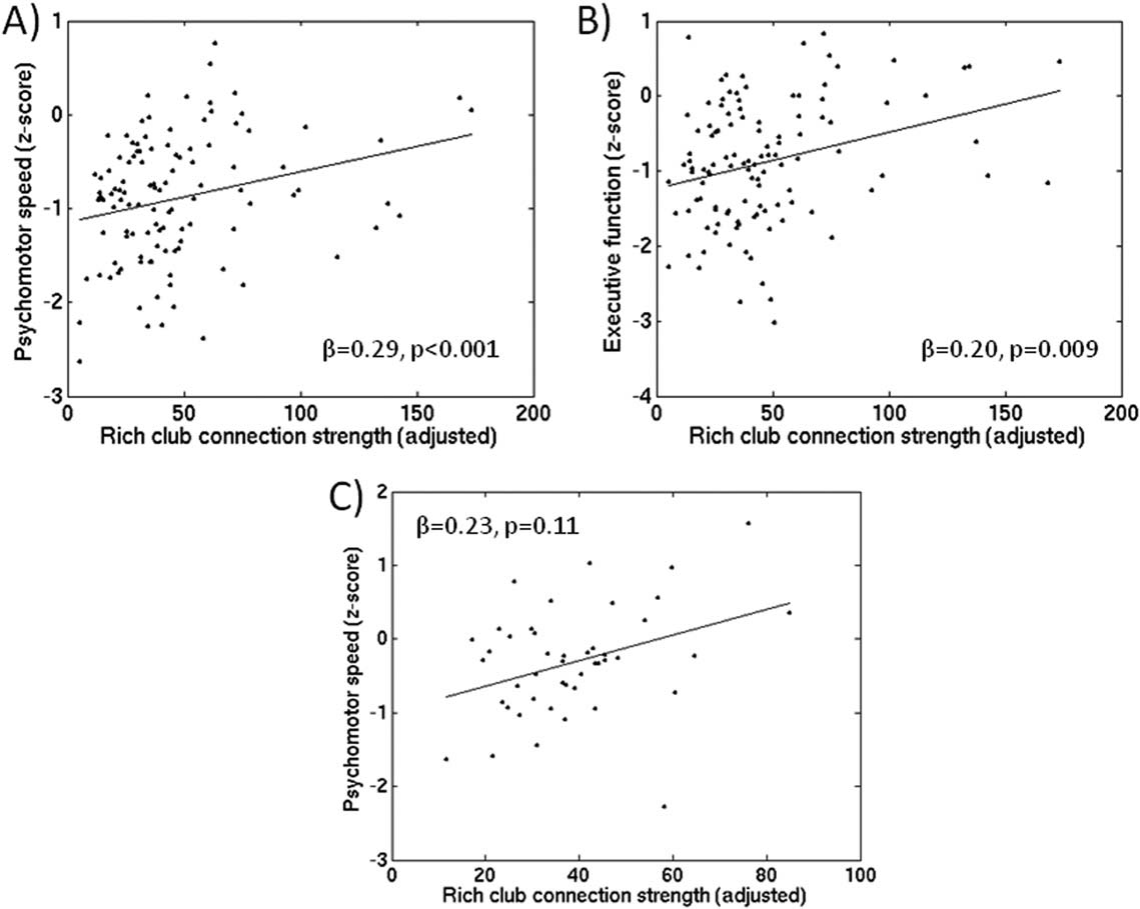
Scatterplots showing the relation between rich club connection strength and cognitive function. In the discovery dataset, rich club connection strength was significantly associated with psychomotor speed (A) and executive function (B), adjusted for age, sex and NART‐IQ. In the replication dataset (C), higher rich club connection strength was associated with higher scores on psychomotor speed, adjusted for age, sex and education, comparable to that seen in the discovery dataset, although this did not reach significance.

In the SVD group, higher connection strength of the rich club connections was associated with better scores on processing speed adjusted for age, sex, NART‐IQ (Fig. 8A; *β* = 0.28, *P* < 0.001). Further adjusting for WMH or lacunes reduced the variance explained, but coefficients remained significant (*β* = 0.22, *P* = 0.015; *β* = 0.17, *P* = 0.041, respectively). Higher WMH and higher lacunes were associated with lower scores on processing speed adjusted for age, sex, NART‐IQ (*β* = −0.21, *P* < 0.001, *β* = −0.33, *P* < 0.001, respectively). A significant indirect effect indicated that rich club connection strength mediated the association between WMH and processing speed (*z* = −2.09, *P* = 0.037), while a marginally significant indirect effect was seen for the association between lacunes and processing speed mediated by rich club connection strength (*z* = −1.91, *P* = 0.056). Higher rich club connection strength was associated with better scores on executive function (Fig. 8B; *β* = 0.20, *P* = 0.009), which was also significant after adjusting for WMH (*β* = 0.17, *P* = 0.041). Higher WMH was not associated with lower scores on executive function (*β* = −0.04, *P* = 0.632), while higher lacune count was associated with lower scores on executive function (*β* = −0.27, *P* < 0.001). However, the explained variance of rich club connection strength on executive function was reduced after controlling for lacune count and was not significant (*β* = 0.10, *P* = 0.21) and likewise no mediation effect was observed for the association between lacune count and executive function.

#### Replication dataset

In the SVD cases the direction of effect and coefficient value of the association between rich club connection strength and psychomotor speed were comparable to that seen in the discovery dataset, although this did not reach significance (Fig. 8C; *β* = 0.23, *P* = 0.11); perhaps reflecting the smaller sample size (*n =* 45 due to missing data for psychomotor speed).

## DISCUSSION

This study investigated the white matter connectivity in symptomatic SVD using two independent datasets. We showed that structural networks in patients with SVD were less dense with lower connectivity strength and efficiency in both the discovery and replication datasets. We showed that the reduction in connectivity of the structural network in SVD is preferentially associated with the rich club connectivity and confirmed this finding in a replication dataset of similar patients. Furthermore, in SVD patients, lower rich club connectivity was associated with lower scores on cognitive function. These results provide empirical evidence for the involvement of the abnormal rich club phenomenon in SVD, which might contribute to the development of cognitive impairment in patients with SVD.

The rich club brain regions are highly connected regions that are also efficiently interconnected with each other [Van den Heuvel and Sporns, 2011]. Among others superior frontal gyrus, precuneus, thalamus and putamen, were identified in both datasets as rich club nodes, consistent with previous studies [Kim et al., 2014; Van den Heuvel and Sporns, 2011]. Due to their central position in the topology of the network, the connections among these rich club nodes might have an important role in the efficient integration of information processing among distant brain regions (i.e., global efficiency of the network) [Lawrence et al., 2014; Van den Heuvel et al., 2012; Van den Heuvel and Sporns, 2011]. Damage specifically to the rich‐club connections seems to have more severe impact on global efficiency than random damage to the network [Van den Heuvel and Sporns, 2011]. In the context of cognitive functions and network, several studies have shown that network efficiency (reflecting the integration over the whole brain‐network) is strongly related to cognition [Giessing et al., 2013; Van den Heuvel et al., 2009; Wen et al., 2011]. Rich club organisation might be specifically relevant for cognitive functions, such as information processing speed, that depend on a distributed network of brain regions and thus are more dependent on efficient integrative network processing [Bassett et al., 2009; Crossley et al., 2014; Dehaene and Changeux, 2011; Zalesky et al., 2012]. As previously shown, widespread reduced white matter connectivity is present in SVD patients [Lawrence et al., 2014] (see also Fig. 6) and global network efficiency was related to cognitive functions [Lawrence et al., 2014; Reijmer et al., 2015; Tuladhar et al., 2015b]. Extending these findings, this study shows that the reduced white matter connectivity in symptomatic SVD patients is disproportionally concentrated among the rich club nodes rather than a generalized reduction of the white matter connectivity. This suggests that the reduced network efficiency in SVD might be related to the disruption of rich club organization of the brain.

The association between the rich club connection strength and cognitive impairment provides evidence for a possible link between the rich club (dis)organisation and the occurrence of cognitive impairment in symptomatic SVD. This is further corroborated by a recent study showing that central network connections, which were based on edge betweenness centrality, were related to cognitive impairment in SVD [Reijmer et al., 2016]. Although network centrality does not directly measure rich club organisation, the connections in the rich club are likely to be among the most central suggesting these independent findings provide convergent evidence. In our study, rich club connection strength mediated the association between WMH and processing speed. In contrast, lacunes had a direct effect on cognition, probably due to their strategic location with damage to an essential pathway. In the replication cohort, the strength of the association between rich club connection strength and psychomotor speed was similar to that in the discovery dataset, but was not statistically significant. This is probably due to the relatively low sample size (*n =* 45). An important question for future research is the time course of network disruption in SVD. It might be that impairment of the rich club connections occurs preferentially with secondary mild impairments of feeder/peripheral connections. Another possibility is that impaired feeder or peripheral connections might be present in the early stages of the disease without evident clinical symptoms, until the rich club connections are affected due to the progression of the disease which then produces clinical overt symptoms [Crossley et al., 2014]. Future longitudinal studies—preferably with inclusion of participants with early‐stage SVD—are warranted to further delineate the relationship between white matter connectivity and cognitive impairment in SVD patients.

Several studies have shown that rich club network is also altered in other brain disorders including neuropsychiatric disorders, such as schizophrenia [Collin et al., 2014a; Van den Heuvel et al., 2013] and autism spectrum [Ray et al., 2014; Watanabe and Rees, 2015]. With respect to our findings in SVD, some evidence have been found for cerebral microvascular abnormality in neuropsychiatric disorders [Hanson and Gottesman, 2005]. An intriguing question is whether the vascular damage might in part be linked to the rich club disorganisation in psychiatric disorders. Furthermore, it was worth noting that several recent studies have shown that stronger rich club connectivity was associated with better cognitive performances in both healthy and psychiatric participants [Baggio et al., 2015; Collin et al., 2016], which further support the link between rich club formation and cognition.

There are several reasons why the rich club organisation might be particularly vulnerable in SVD patients. SVD is characterized by the presence of WMH and lacunes of presumed vascular origin [Wardlaw et al., 2013]. The most common site of WMH is the periventricular white matter (Fig. 1), affecting among others the long association fibres, and the most common sites of lacunes (among others) are the basal ganglia and thalamus [Benjamin et al., 2014]. These regions are commonly found to be involved in the rich club organisation in structural networks [Owen et al., 2015] and thus damage to these regions or white matter tracts among these regions may produce disproportionate disturbance in the rich club organisation. Although the number of rich club connections is small, the widespread nature of white matter damage in SVD may have a greater overall impact on these connections due to the spatial embedding of the connections. In support of this argument, we found that higher WMH volume was significantly associated with lower connection strength of the rich club connections (*P* < 0.001) and that the number of lacunes was marginally significant with the rich club connection strength (*P* = 0.064), while WMH or lacunes was not associated with non‐rich club connections (Table 3).

**Table 3:** Correlation between SVD markers and connection strength in discovery dataset

	Rich club connection strength	Feeder/peripheral connection strength
SVD group		
WMH	−0.38 (*P* < 0.001)	0.11 (*P* = 0.235)
Lacunes	−0.17 (*P* = 0.064)	−0.09 (*P* = 0.338)

Data are Pearson's correlation. The connection strength for the rich club, feeder and peripheral connections are corrected for the overall connection strength. SVD, small vessel disease; WMH, white matter hyperintensity.

An alternative explanation arises from physiology: it has been argued that the rich club nodes are biologically costly. Rich club nodes tend to have a high rate of metabolic activity and are connected by costly long connections [Alexander‐Bloch et al., 2013; Collin et al., 2014b; Vaishnavi et al., 2010; Van den Heuvel and Sporns, 2011]. Tracts running longer physical distances tend to require higher level of energy consumption [Bullmore and Sporns, 2012]. As damage in SVD is caused by ischemia, the high metabolic rich club connections may be particularly affected. In line with this, previous results in SVD identified the sub‐network of most impaired white matter connectivity which was characterized by involvement of inter‐hemispheric and long‐range association tracts, many of these tracts passed through regions commonly affected by WMH [Lawrence et al., 2014]. In addition, we showed that the short length feeder connections resemble the rich club connections, while the long feeder connections are substantially less affected by SVD (Fig. 7). One reason for this may be the spatial embedding of the connections. By definition feeder connections are connected to the rich club at one end, and thus shorter feeder connections are likely to spend a greater proportion of their length running close to the rich club. SVD effects on the rich club may have a greater impact on the short feeder connections by co‐locality. Alternatively, and from a network perspective, if SVD preferentially affects the rich club first then diffuses out through the network, the shortest feeder connections would be impacted next. Longitudinal research is required to investigate these hypotheses further.

The major strength of this study is the inclusion of an independently acquired dataset, replicating the key case‐control findings in the discovery dataset. This replication shows that the presented findings can generalise between two studies with differing recruitment criteria, MRI protocols and image analysis pipelines.

Several methodological issues and limitations should be considered. Structural networks were created from DTI and deterministic streamlining based on tensor reconstruction model. These techniques are computationally inexpensive and robust in terms of identification of major white matter tracts. However, they are limited by partial volume effects, and identification of white matter tracts in complex white matter architecture [Zalesky and Fornito, 2009]. DTI data were acquired at 1.5 Tesla with a relatively low number of diffusion directions, which limits us from performing sophisticated tractography algorithms that account for crossing fibres. The consistency between the studies with regard to the relation between global efficiency and cognition [Lawrence et al., 2013; Reijmer et al., 2015; Tuladhar et al., 2015b; Van den Heuvel et al., 2009; Wen et al., 2011], and identification of the similar rich club members [Kim et al., 2014; Van den Heuvel and Sporns, 2011], suggests that the whole‐brain tractography approach and network analysis is reliable in SVD. Furthermore, in the discovery dataset we have applied super‐resolution seeding to minimize the impact of low spatial resolution on tract reconstruction [Calamante et al., 2010; Lawrence et al., 2014]. More sophisticated tractography methods (i.e., spherical deconvolution and probabilistic tractography) and construction of the white matter tracts at high‐resolution are needed to verify these findings and might provide more detailed information about the white matter tracts and network architecture of SVD.

**Figure 9:**
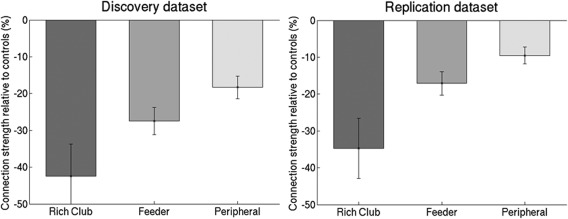
Rich impairment in the discovery and replication dataset using identical set of rich club regions in both datasets. The rich club regions included bilateral superior frontal gyrus, precuneus, superior parietal gyrus and insula, which were previously reported as rich club nodes [Collin et al., 2014a; van den Heuvel et al., 2013]. Using these rich club nodes, we found that SVD patients showed 42.5% reduction in the connectivity strength of the rich club connections (*P* < 0.001, df = 83.8), 27.5% reduction in feeder (*P* < 0.001, df = 92.4) and 18.3% reduction in peripheral connections (*P* < 0.001, df = 99.2) relative to the control group in the discovery dataset. Compared with the control group, SVD patients showed significantly lower ratios for rich club/feeder (*P* < 0.001) and rich club/peripheral connection (*P* < 0.001). In the replication dataset, SVD patients had 34.7% reduction in the connectivity strength of the rich club connections (*P* < 0.001, df = 114.2), 17.1% reduction in feeder (*P* < 0.001, df = 93.8) and 9.5% reduction in peripheral connections (*P* < 0.001, df = 88.7) relative to the control group. Ratios for rich club/feeder and rich club/peripheral connections were significantly reduced in SVD group (*P* = 0.009; *P* < 0.001, respectively).

Another limitation is the differences in preprocessing methodology between the two datasets, which reduce the control over experimental factors and exclude a systematic investigation of different processing methods. Such differences might be expected to reduce similarities between findings from the two datasets; however we found very similar patterns of rich club disorganisation in the two cohorts of SVD cases. Another consideration is the selection of the rich club nodes: we base our main findings on a rich club defined from the 8 nodes with the highest average degree (across both patient and control participants). Other methods can be used and were evaluated in this study: identification of rich club nodes based on a group‐averaged network [De Reus and Van den Heuvel, 2013a] and by calculation on individual networks. The optimal procedure for the construction of a group‐averaged network and determining adequate threshold in elderly subjects with neurological disease is currently unclear. Individual‐level definition also has potential bias, in that patients have fewer connections and less interconnected hubs, and therefore possibly investigating different regions across the subject groups. Although these supplementary methods, the additional analysis of rich club selection [including analyses using identical set of rich club nodes in both datasets (Fig. 9)], different weighting procedures and different number of rich club nodes showed similar results to the main analysis technique, further studies are important to investigate how the definition of the rich club influences rich club properties, especially in elderly patients. As these results are based on cross‐sectional data they are limited in that no direct causal relationship can be inferred between the impaired rich club organisation and cognitive impairment. Finally, in the replication dataset the number defined as SVD cases was relatively low (*n =* 49), which reduced statistical power for testing within group associations with cognitive performance.

SVD represents a broad phenotype ranging from asymptomatic WMH in community populations to symptomatic patients with severe radiological disease and symptomatic lacunar stroke and/or vascular cognitive impairment. We chose a group with a well‐defined phenotype, namely radiologically confirmed lacunar stroke and confluent WMH. It is likely similar findings will apply to other phenotypes of SVD but this needs confirming in further studies.

In conclusion, this study provides a novel perspective on how SVD disrupts brain network organisation. Rich club connections are a fundamental motif in brain networks and are thought to be key for the integration of information among distributed network modules. In SVD the connections of the rich club were disproportionally impaired and associated with cognitive impairment. Further longitudinal research is needed to examine how and whether the changes of white matter connectivity, including the investigation of the rich club properties, are related to clinical deterioration in SVD patients.
